# Whole-Genome Sequence of the Endophytic Actinacidiphila bryophytorum Strain DS3, Isolated from the Roots of the Medicinal Plant Dysosma versipellis

**DOI:** 10.1128/mra.01171-22

**Published:** 2023-02-02

**Authors:** Qian Huang, Luchun Wu, Xinyi Xu, Guihua Tian, Hang Zou, Xingyong Yang

**Affiliations:** a College of Pharmacy, Chengdu University, Chengdu, China; b Chongqing Qiantai Biopharmaceutical Co. Ltd., Chongqing, China; c Antibiotics Research and Re-evaluation Key Laboratory of Sichuan Province, Chengdu University, Chengdu, China; University of Arizona

## Abstract

We report the whole-genome sequence of the endophytic Actinacidiphila bryophytorum strain DS3, which was isolated from the roots of the medicinal plant Dysosma versipellis. The DS3 strain genome consists of a chromosome of 8,265,668 bp, with a GC content of 72.47%, including 7,121 coding sequences.

## ANNOUNCEMENT

Actinomycetes are known for their ability to produce a variety of structurally complex but functionally important, biologically active, natural products (1). *Actinacidiphila* strains are Gram-positive, filamentous bacteria of the order *Streptomycetaceae*, with more than 10 described species, and are a common source of biologically active molecules, such as antibiotics (2). In addition to antibiotics, *Actinacidiphila* strains can produce various extracellular hydrolytic enzymes and other active substances that are desirable for suppressing diverse plant pathogens (3).

Here, the endophytic Actinacidiphila bryophytorum strain DS3 was isolated from the roots of a medicinal plant, Dysosma versipellis, in the medicinal botanical garden of the Chongqing Institute of Medicinal Plant Cultivation in China (29°7′56″N, 107°12′10″E). Isolation of strain DS3 was conducted on Gause’s medium number 1 (4) plates using the Coombs’ method (5). Briefly, the roots were washed in turn with 3% NaOCl for 60 s, 75% ethanol for 30 s, and sterilized water for 20 s (three times), cut into 0.5-cm pieces, and distributed onto Gause’s medium plates for incubation at 26°C for 1 week. The single colony of this strain was cultured in Gause’s medium at 30°C in a shaker at 200 rpm for 5 days, and the cells were collected by centrifugation at 10,000 × *g* for 5 min. Genomic DNA of DS3 was extracted using the Wizard genomic DNA purification kit (Promega) according to the manufacturer’s protocol. Illumina sequencing libraries were prepared using the NEXTflex rapid DNA-sequencing kit and then were used for paired-end Illumina sequencing (2 × 150 bp) on a NovaSeq 6000 platform (Illumina, San Diego, CA, USA). For Nanopore sequencing, approximately 10 μg of genomic DNA was isolated and size selected using BluePippin (Sage Science, Beverly, MA, USA) and processed according to the 1D ligation sequencing kit (SQK-LSK109) protocol. The library was loaded onto a R9.4 flow cell and sequenced using a PromethION DNA sequencer (Oxford Nanopore Technologies [ONT], Oxford, UK) for 48 h. The raw Illumina sequencing reads generated from the paired-end library were subjected to quality filtering using fastp v0.23. Nanopore reads were extracted, base called, demultiplexed, and trimmed using ONT Guppy, with a minimum Q score cutoff value of 7. After the raw data were filtered, a total of 156,896 reads (*N*_50_, 27,912 bp; *N*_90_, 6,375 bp) with an average length of 14,499 bp (total of 2,274,854,698 bp) were obtained. The clean short and long reads were then coassembled to construct complete genomes using Unicycler v0.4.8 (6). Finally, Unicycler used Pilon v1.22 (7) to polish the assembly using short-read alignments, reducing the rate of small errors. Glimmer (http://ccb.jhu.edu/software/glimmer/index.shtml) and Prodigal v2.6.3 were used to predict coding sequences (CDSs) (8). The predicted CDSs were annotated from the nonredundant protein (NR), Swiss-Prot, protein family (Pfam), Gene Ontology (GO), Cluster of Orthologous Groups (COG), and Kyoto Encyclopedia of Genes and Genomes (KEGG) databases using sequence alignment tools BLAST v2.3.0, DIAMOND v0.8.35, and HMMER v3.1b2. The tRNA-scan-SE v2.0 (9) and Barrnap v0.9 (https://github.com/tseemann/barrnap) predictors were used for tRNA and rRNA prediction, respectively. Infernal software (http://eddylab.org/infernal) and the Rfam database (https://rfam.xfam.org) were used for small RNA (sRNA) prediction. In addition, carbohydrate-active enzymes (CAZymes) were annotated with the CAZymes database (http://www.cazy.org), and secondary metabolite biosynthetic gene clusters (BCGs) were annotated with the antiSMASH website (http://antismash.secondarymetabolites.org/#!/start). Default parameters were used for all software unless otherwise specified.

The genome of A. bryophytorum DS3 consists of a linear chromosome of 8,265,668 bp, with a GC content of 72.47%, containing 6 tRNAs, 18 rRNAs, 33 sRNA genes, 104,093-bp tandem repeats, and 7,121 CDSs. Of these predicted CDSs, 7,027 genes, 4,567 genes, 5,680 genes, 5,380 genes, 4,070 genes, and 1,837 genes were mapped and annotated using the NR, Swiss-Prot, Pfam, COG, GO, and KEGG databases, respectively. A total of 369 CAZymes were identified, including 25 auxiliary activities, 12 carbohydrate-binding modules, 67 carbohydrate esterases, 193 glycoside hydrolases, 56 glycosyl transferases, and 16 polysaccharide lyases. Using antiSMASH software, 31 secondary metabolite BCGs were predicted, including 7 polyketide synthetases, 7 nonribosomal peptide synthetases, 1 siderophore, 3 terpenes, and multiple antibiotic gene clusters (Table 1). Overall, the complete genome sequence of the DS3 strain provides valuable data on endophytic *Actinacidiphila* organisms and potential antimicrobial mechanisms of strain DS3.

**TABLE 1:** Secondary metabolite BGCs in Actinacidiphila bryophytorum DS3

BCG type^*a*^	Most similar cluster	Similarity (%)
NRPS-like	5-Isoprenylindole-3-carboxylate β-d-glycosyl ester	28
NRPS-like	Lividomycin	10
NRPS-1	Scabichelin	100
NRPS-2	AmfS	80
NRPS-3	Furaquinocin B	21
NRPS-4	Microtermolide A	13
NRPS-5	Paramagnetoquinone 1/paramagnetoquinone 2	10
PKS-like	Chalcomycin A	4
T3PKS-1	Alkylresorcinol	100
T3PKS-2	CDA1b/CDA2a/CDA2b/CDA3a/CDA3b/CDA4a/CDA4b	17
T2PKS-1	Spore pigment	83
T2PKS-2	Sch-47554/Sch-47555	38
T2PKS-3	Saprolmycin E	47
T1PKS	Armeniaspirols	34
Lanthipeptide-1	Kanamycin	2
Terpene-1	Pristinol	100
Terpene-2	Hopene	69
Terpene-3	Ebelactone	8
Phenazine	Endophenazine A/endophenazine B	27
LAP	Meilingmycin	3
Siderophore	Ficellomycin	3
TfuA-related	Thiovarsolin A/thiovarsolin B/thiovarsolin C/thiovarsolin D	33
Melanin	4-Hydroxy-3-nitrosobenzamide	50

a NRPS, nonribosomal polypetide synthetase; PKS, polyketide synthetase; LAP, linear azol(in)e-containing peptides.

### Data availability.

The complete genome sequence of Actinacidiphila bryophytorum strain DS3 was deposited in NCBI GenBank under accession number CP103871 (BioProject accession number PRJNA873045 and BioSample accession number SAMN30478166). The raw data were deposited in the NCBI Sequence Read Archive (SRA) under SRA accession number SRR22779143.
